# Undrained stability of braced excavations in clay considering the nonstationary random field of undrained shear strength

**DOI:** 10.1038/s41598-023-40608-5

**Published:** 2023-08-16

**Authors:** Weeradetch Tanapalungkorn, Wittawat Yodsomjai, Suraparb Keawsawasvong, Thanh Son Nguyen, Weeraya Chim-Oye, Pornkasem Jongpradist, Suched Likitlersuang

**Affiliations:** 1 Centre of Excellence in Geotechnical and Geoenvironmental Engineering, Department of Civil Engineering, Faculty of Engineering, Chulalongkorn University, Bangkok, 10330 Thailand; 2 Construction Innovations and Future Infrastructures Research Center, Department of Civil Engineering, Faculty of Engineering, King Mongkut’s University of Technology Thonburi, Bangkok, 10140 Thailand; 3 Research Unit in Sciences and Innovative Technologies for Civil Engineering Infrastructures, Department of Civil Engineering, Faculty of Engineering, Thammasat School of Engineering, Thammasat University, Pathumthani, Thailand; 4 Department of Civil Engineering, Faculty of Engineering, Thammasat School of Engineering, Thammasat University, Pathumthani, Thailand

**Keywords:** Engineering, Mathematics and computing

## Abstract

The basal heave stability of supported excavations is an essential problem in geotechnical engineering. This paper considers the probabilistic analysis of basal heave stability of supported excavations with spatially random soils by employing the random adaptive finite element limit analysis and Monte Carlo simulations to simulate all possible outcomes under parametric uncertainty. The effect of soil strength variability is investigated for various parameters, including the width and depth of the excavation ratio, strength gradient factor, and vertical correlation length. Probabilistic basal stability results have also been employed to determine the probability of design failure for a practical range of deterministic factors of safety. Considering probabilistic failure analysis, the more complete failure patterns caused by the various vertical correlation length would decrease the probability of design failure. There are different tendencies between the probability of design failure at the same safety factor with various vertical correlation lengths. These results can be of great interest to engineering practitioners in the design process of excavation problems.

## Introduction

Population growth has led to an increase in demand for city infrastructure, especially in megacities worldwide. To cope with the growing demand, effective use of underground space is essential to alleviate land scarcity. The stability of the excavation system is crucial because of safety and reliability considerations, with basal heave stability being a significant concern contributing to braced excavation system failure. Previous studies have focused mostly on excavation problems related to wall movements and lateral earth pressures. The classic stability solutions of supported excavations have been assessed using a limit equilibrium approach proposed by Terzaghi^1^ and Bjerrum and Eide^2^. Later researchers such as Goh^3^, Faheem et al.^4^, and Goh et al.^5^ investigated the basal heave stability problems under plane strain conditions using the finite element method (FEM). Meanwhile, stability studies of supported excavations have expanded to three-dimensional problems for rectangular and circular excavations using FEM (e.g., Cai et al.^6^; Faheem et al.^7^; Goh^8^; and Goh^9^).

The finite element limit analysis (FELA) was carried out by Ukritchon et al.^10^; Keawsawasvong and Ukritchon^11^; Chen et al.^12^; Lai et al.^13^; and Kounlavong et al.^14^ to investigate the basal stability of supported planar and circular excavations with full bracing. FELA is a powerful numerical technique based on plastic limit analysis theory, which can provide upper and lower-bound estimations of the actual collapse load^15^. Early versions of FELA used linear programming, while recent developments utilise nonlinear programming formulations^16–21^. However, limited research on utilising adaptive finite element limit analysis (AFELA) to examine fully braced excavations in soils with spatial variability can be found. Previous studies of plane strain fully braced excavations using FELA relied on deterministic analysis. The current study aims to contribute to the existing knowledge by addressing this problem from a broader perspective, particularly in spatially random clays.

Soil parameters, namely cohesion, frictional angle, and soil unit weight are widely found to differ greatly from one site to another. Typically, they have geographically random fields. Probabilistic analysis has also been employed to investigate the effects of geographical variability and form better conclusions on a project’s eventual results. Several studies on the random field have been adopted in many geotechnical problems, such as slope stability^22–25^, embankment^26^ and deep excavation^27^. The undrained shear strength is generally handled as a random field with a log-normal distribution and a spatial correlation length for undrained stability concerns^28^. Griffiths and Fenton^29^ and Griffiths et al.^30^ conducted initial investigations that interpreted the bearing capacity of a strip footing from a series of Monte Carlo simulations statistically by incorporating a Cholesky decomposition method with midpoint discretisation to integrate the lognormally distributed undrained shear strengths to an elastoplastic displacement-based finite element analysis^31^. The random finite element method (RFEM) is a well-known approach similar to the earlier described technique, which Griffiths and Fenton^32^ and Griffiths et al.^33^ utilised to study the effects of spatial variability on slope dependability. Zhu et al.^34^ applied the RFEM to study the limit load of a shallow passive trapdoor in clay by considering the influence of strength variability of spatially random clay.

The random finite element limit analysis (RFELA) based on the numerical upper bound (UB) and lower bound (LB) theorem of plasticity that incorporates the stochastic spatial variability of undrained shear strength was employed to explore problems of slope reliability (e.g., Kasama and Zen^35^; Huang et al.^36^; Halder and Chakraborty^37^), probabilistic passive resistance of retaining walls (e.g., Krishnan and Chakraborty^38^) and probabilistic bearing capacity, (e.g., Kasama and Whittle^39^; Huang et al.^40^; Li et al.^41^; Kasama et al.^42^). However, those studies employed a uniform mesh in which all element sizes are constant in the modelled domain. Using a uniform mesh for computations has a clear disadvantage in that refining the mesh may be necessary to obtain a suitable final mesh. This process can also be time-consuming, particularly when attempting to precisely estimate the solution’s accuracy.

The recent development of random adaptive finite element limit analysis (RAFELA) developed by Ali et al.^43^ was the first to propose using RAFELA for slope stability and bearing capacity problems. Using RAFELA, tight bounds on probabilistic results in each simulation can be obtained with a very high level of accuracy. The details of FELA with the adaptive mesh refinement can be found in Lyamin et al.^44^. A growing body of literature has recognized the importance of RAFELA for the probabilistic analysis in geotechnical stability, such as unlined tunnels (e.g., Ali et al.^45^; Ali et al.^46^; Wu et al.^47^), slope stability^25^, strip footings lying on voids^48^, inclined loaded strip footings near cohesive slopes^49^, and risk assessment of earth dam^50^. The latest development of the RAFELA technique can be found in the OptumG2 FELA software^21^. There were limited studies focusing on the probabilistic analysis of basal heave stability of fully braced excavations in spatially variable clays^51,52^.

This study investigates the undrained stability of fully braced excavations in spatially variable soils by utilising advanced RAFELA. This work aims to measure the effects of geographical variability and geometrical parameters on the mean stability number of fully braced excavation and the failure probability. A reasonable set of parameters was chosen for the parametric studies, and the likelihood of probabilistic failure (*P*_*f*_) with different influential parameters was supplied for practical usage. In addition, Monte Carlo simulations (MCs) were used to illustrate chosen instances of the related failure processes and to provide a deeper understanding and explanation of how random fields could affect excavation failures.

## Problem definition

**Figure 1:**
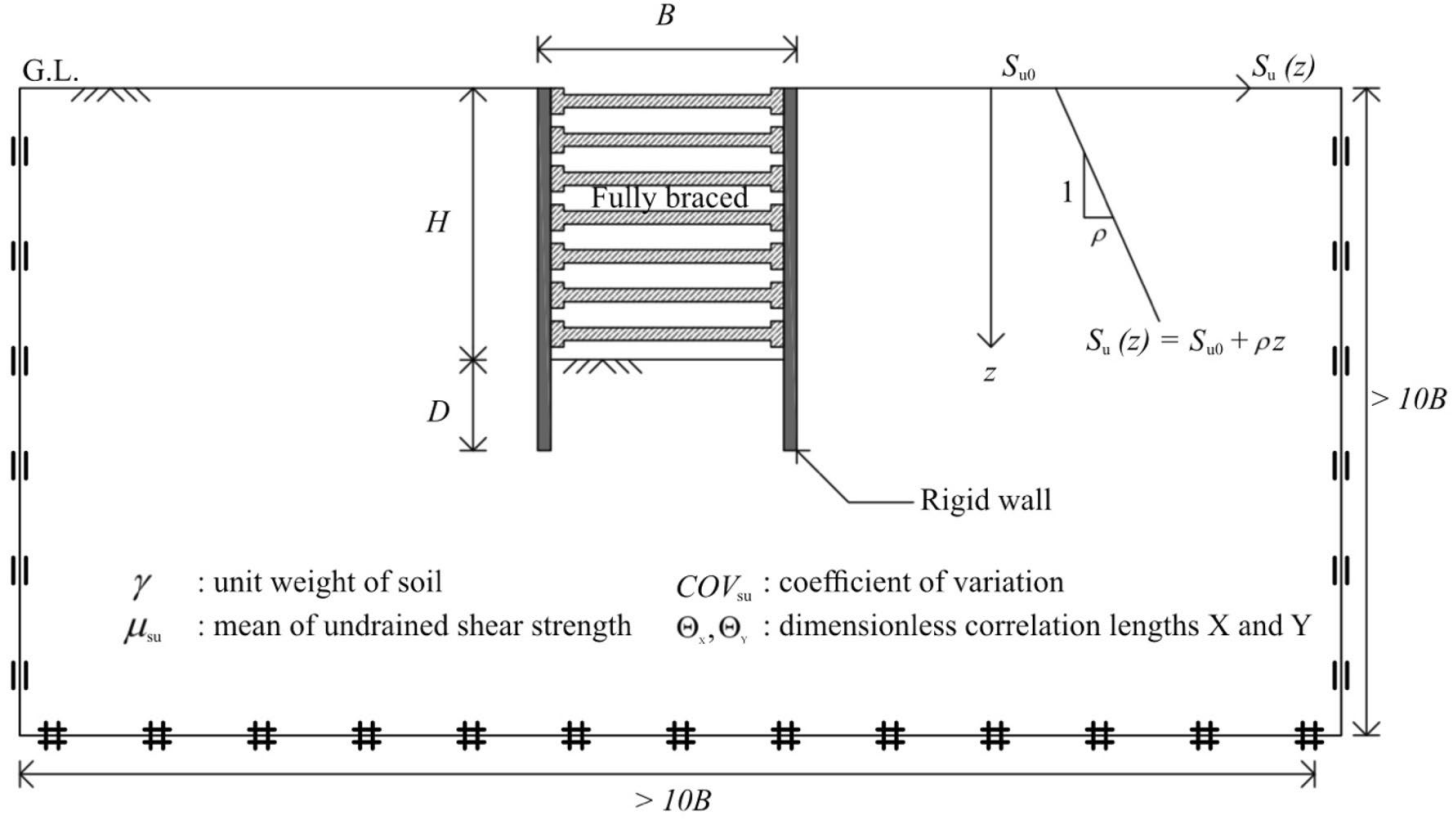
Problem definition.

In this study, a braced excavation in spatially random clay with a linear increase of strength with depth is defined for deterministic and stochastic analyses and shown in Fig. 1. The excavation is under plane strain conditions and has a width (*B*) and depth (*H*) of excavation and the depth of embedment (*D*). The clay is considered a rigid-perfectly plastic Tresca material with a mean value of undrained shear strength (*μ*_su0_) at a depth *z* = 0 and linear strength gradient *ρ*. As a result, the mean value of undrained shear strength at any depth can be written as *μ*_su_ = *μ*_su0_ + *ρz*, which is a function of depth. The linear function of undrained shear strength with depth was originally proposed by Bishop^53^. By defining the linear function that relates the undrained shear strength to the random field, the function can take the random value as input and return a corresponding undrained shear strength value. As a result, a non-stationary random field can be generated. More information regarding the non-stationary random field can be found in Yi et al.^54^ and Liu et al. ^55^.

The constant unit weight (*γ*) is the objective variable (or output) for the braced excavation in spatially random clay. For deterministic analysis, the stability number (*N*) of this excavation problem in plane strain conditions is the normalised term of the unit weight^10^, which can be expressed as follows:

1 $$N=\frac{\gamma H}{{\mu }_{\text{su}0}}.$$

This stability number results in AFELA, which can be known as a function of the normalised width of excavation (*B/H*), the normalised depth of embedment (*D/H*), the strength gradient ratio (*ρH/μ*_su0_), and some dimensionless coefficients describing the inherent spatial variability of soil concisely. Note that the definition of the stability number (*N*) and other input non-dimensional parameters are based on the previous studies by Ukritchon et al.^10^; Keawsawasvong and Ukritchon^11^; and Kounlavong et al.^14^. The latter will be described extensively in Section "Random field theory".

## Random field theory

This study considers clay’s undrained shear strength (*s*_u_) as the spatial variability for probabilistic investigations. The spatial variability of soil properties is assigned to any location in a domain known as random fields. According to the random field theory^56–62^, parameters *CL*_x_ and *CL*_y_ are carried out to define dimensional spatial correlation lengths in horizontal and vertical directions to capture the scale of fluctuations of random soils. These parameters represent a characterisation of spatial variability of *s*_u_ in the soil domain. A large dimensional spatial correlation length yields the smoothly varying field of random soils, whereas a low one makes a ragged random field. Every point in the random field becomes independent when the dimensional spatial correlation length approximates zero. In the random field context, *CL*_x_ and *CL*_y_ are generally incorporated through a correlation function *ρ* which can be assumed by the exponential function as shown in Eq. (2a).

2a $$\rho ({\tau }_{\text{xij}},{\tau }_{\text{yij}})=\text{exp}\left(-\frac{{\tau }_{\text{xij}}}{{CL}_{\text{x}}}-\frac{{\tau }_{\text{yij}}}{{CL}_{\text{y}}}\right),$$

where ${\tau }_{\text{xij}}$ and ${\tau }_{\text{yij}}$ are the absolute horizontal and vertical distances between two discrete points, respectively; *CL*_x_ and *CL*_y_ are the horizontal and vertical correlation lengths, respectively. The dimensionless correlation lengths Θ_X_ and Θ_Y_ are defined as follows:

2b $${\Theta }_{\text{X}}=\frac{{CL}_{\text{x}}}{H},$$

2c $${\Theta }_{\text{Y}}=\frac{{CL}_{\text{y}}}{H},$$

where *H* is the depth of excavation.

In this paper, Karhunen–Loeve (K–L) expansion method is adopted for modelling random fields because of the advantages of exponential covariance. According to K–L expansion, the analytical solution of the eigenvalue problem for an exponential function (Eq. 2) is used to generate a continuous random field. The details of the procedures can be found in Cho^63^.

The log-normal distributions commonly produce positive variables without a negative random value of *s*_u_. Note that the natural log (*e* = 2.718) of random variables from a normal distribution curve is carried out in the log-normal distribution. By adopting the probability density function (*PDF*)^39,43^, the log-normal distribution of the undrained shear strength of clay can be expressed in Eqs. (3) to (6).

3 $$f\left(x\right)=\frac{1}{x{\sigma }_{\text{lnx}}\sqrt{2\pi }}\text{exp}\left[-\frac{1}{2}{\left(\frac{\text{ln}x-{\mu }_{\text{lnx}}}{{\sigma }_{\text{lnx}}}\right)}^{2}\right]\text{for }x>0,$$

4 $$\text{where}\,{\sigma }_{\text{lnx}}=\sqrt{\text{ln}\left(1+{COV}^{2}\right),}$$

5 $$COV=\frac{{\sigma }_{\text{su}0}}{{\mu }_{\text{su}0}},$$

6 $${\mu }_{\text{lnx}}=\text{ln}\,{\mu }_{\text{x}}-\frac{1}{2}{\sigma }^{2}\text{ln}x.$$

The coefficient of variation (*COV*) is a practical parameter for describing the inherent variation of soil properties in the field^56–62^. To obtain the cumulative distribution function (*CDF*) for a continuous random variable, the probability density function (*PDF*) in Eq. (2) is integrated, where the expression of the *CDF* is shown in Eq. (7).

7 $$F\left(x\right)=\frac{1}{2}\text{erfc}\left(-\frac{\text{ln}x-{\mu }_{\text{lnx}}}{{\sigma }_{\text{lnx}}\sqrt{2}}\right)$$

where erfc is the complementary error function^47,48^.

The probability of design failure (*P*_*f*_) for a practical design application with a factor of safety is also an interesting issue. In this work, the failure is defined to occur when the calculated result of the stability number from the probabilistic analysis with random field theory (*N*_ran_) is less than the deterministic one (*N*_det_) with an appropriate factor of safety (*FS*) as follows:

8 $$\text{Failure is defined if }{N}_{\text{ran}}<\left({N}_{\text{det}}/FS\right)$$

Therefore, the probability of design failure (*P*_*f*_) can be defined as the probability that the number of calculated random stability numbers (*N*_ran_) is less than the “factored” deterministic value (i.e., *N*_det_/*FS*) over the number of realisations or the number of Monte-Carlo simulations (*N*_c_). *N*_c_ can be set as 1,000 times to ensure reliable results of stochastic analysis. Note that this number of 1,000 times the MCs is selected based on several previous studies (e.g., Kasama and Zen^35^; Kasama and Whittle^39^; Huang et al.^36^; Huang et al.^40^; Li et al.^41^; Ali et al.^43^; Ali et al.^45^; Ali et al.^46^; Wu et al.^47^; Wu et al.^48^). The probability of design failure can be approximated as follows:

9 $${P}_{f}=P\left[{N}_{\text{ran}}<\left(\frac{{N}_{\text{det}}}{FS}\right)\right],$$

where *P*_*f*_ is the probability of design failure for a given value of *FS*.

## Random adaptive finite element limit analysis

**Figure 2:**
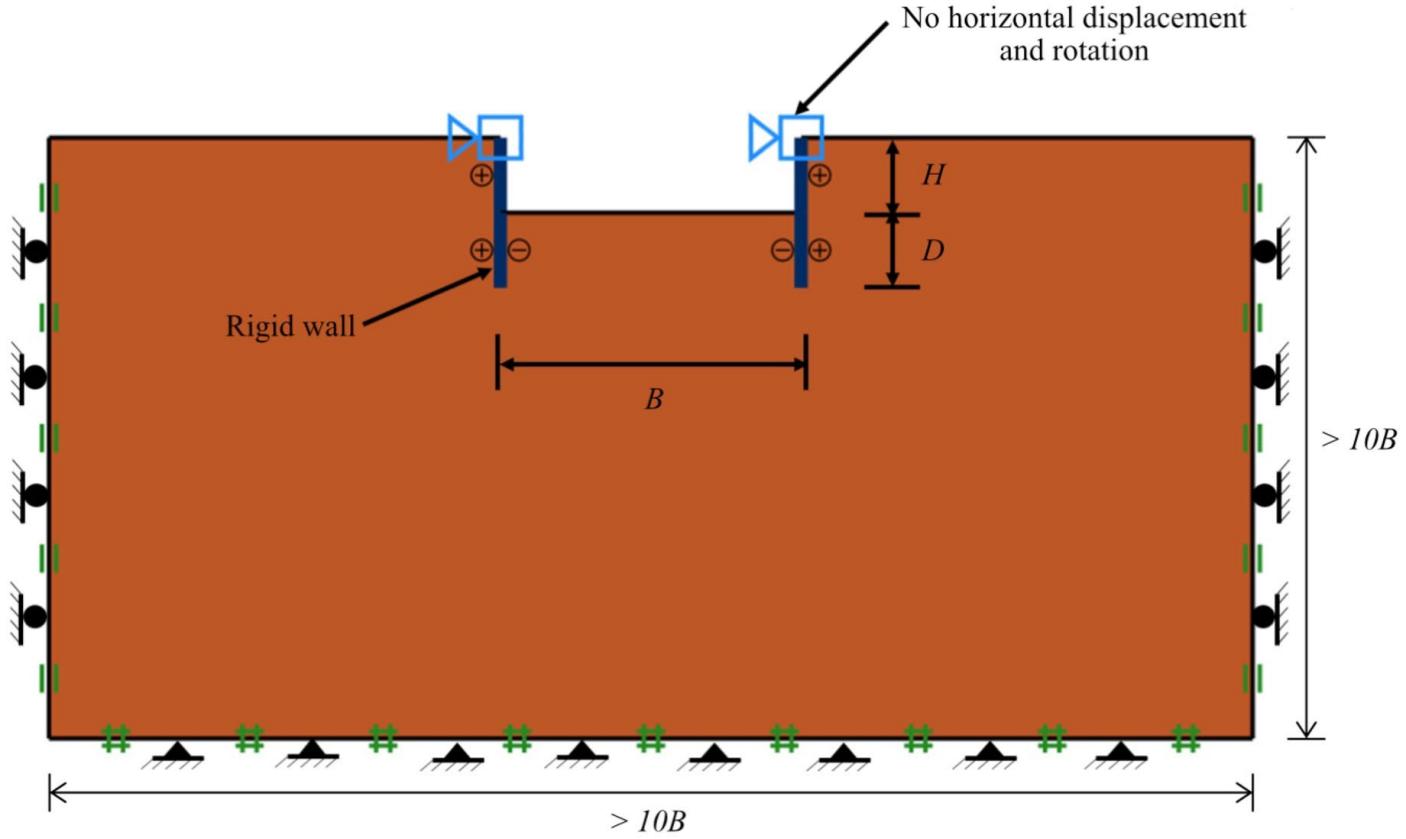
A numerical model in OptumG2.

A numerical model of a braced excavation in the spatial variability of soil properties in OptumG2 is shown in Fig. 2. The boundary dimensions were defined to be at least 10 times of the width of excavation (*B*) in both vertical and horizontal as illustrated in Fig. 2. In the x-direction, the far vertical sides of the model are constrained. The bottom boundary domain is fixed in the x- and y-directions and the top ground surface and excavation area are free-moving surfaces. The wall is modelled by rigid plate elements, where the top of the plate boundary is activated by setting the wall to no horizontal moments and rotations to simulate the fully braced wall. All numerical models are rigorously built to ensure that the domain is adequately broad to prevent the boundary impact to achieve appropriate answers. The objective function in this study is the maximum unit weight of clay that results in the basal heave and the occurrence of the failure. The parameters arrangement for stochastic analysis are listed in Table 1.

**Table 1:** Parameters arrangement for stochastic analysis.

Fixed parameters for all cases	
Coefficient of variation	*COV*_su_ = 25%, 60%
Horizontal correlation length	Θ_x_ = 50.0
Depth of excavation	*D* = 1.0 m
Mean undrained shear strength	*μ*_su0_ = 100 kN/m^2^
Number of Monte-Carlo runs	1000
Number of elements	3000
Number of mesh refinement	3
Variable parameters	
Vertical correlation length	Θ_Y_ = 0.125, 0.25, 0.50, 1.00, 2.00, 4.00
Width of excavation ratio	*B/H* = 0.25, 0.5, 1.0, 2.0, 4.0
Depth of excavation ratio	*D/H* = 0.25, 0.5, 0.75, 1.0
Strength gradient factor	*ρH/μ*_su0_ = 0, 1.0, 2.0
Factor of safety	*FS* = 1.0, 1.2, 1.4, 1.5, 1.6, 1.8, 2.0, 3.0

The adaptivity approach proposed by Lyamin et al.^44^ is applied to increase the precision of upper and lower-bound solutions by merging adaptive mesh refinement with random finite element limit analysis. More information regarding RAFELA can be found in Ali et al.^43^ The internal dissipation estimated from deviatoric stresses and strain rates (also known as shear power) is employed as the covariate in this adaptivity system. Three repetitions of adaptive meshing were utilised in all numerical simulations of the study, having the initial amount of 1,000 elements to the final amount of 3,000 elements. The Karhunen–Loeve (KL) expansion method is used to build a trustworthy random field for RAFELA.

**Figure 3:**
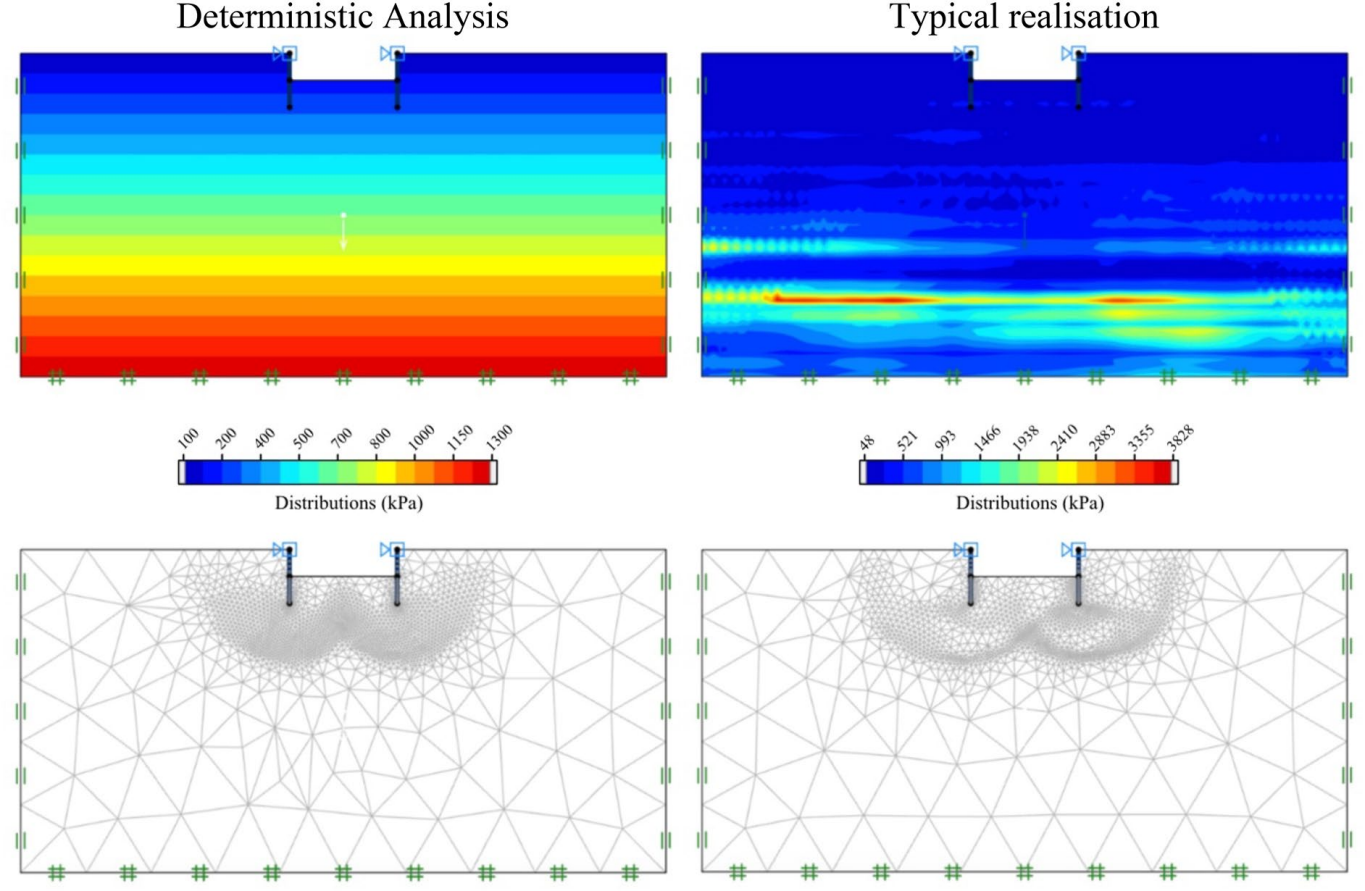
Distribution of *s*_*u*_ and detailed mesh arrangement of proposed AFELA and RFELA for *B/H* = 4.0, *D/H* = 1.0, *ρH/μ*_su0_ = 1.0, Θ_X_ = 50.0, and Θ_Y_ = 1.00.

**Figure 4:**
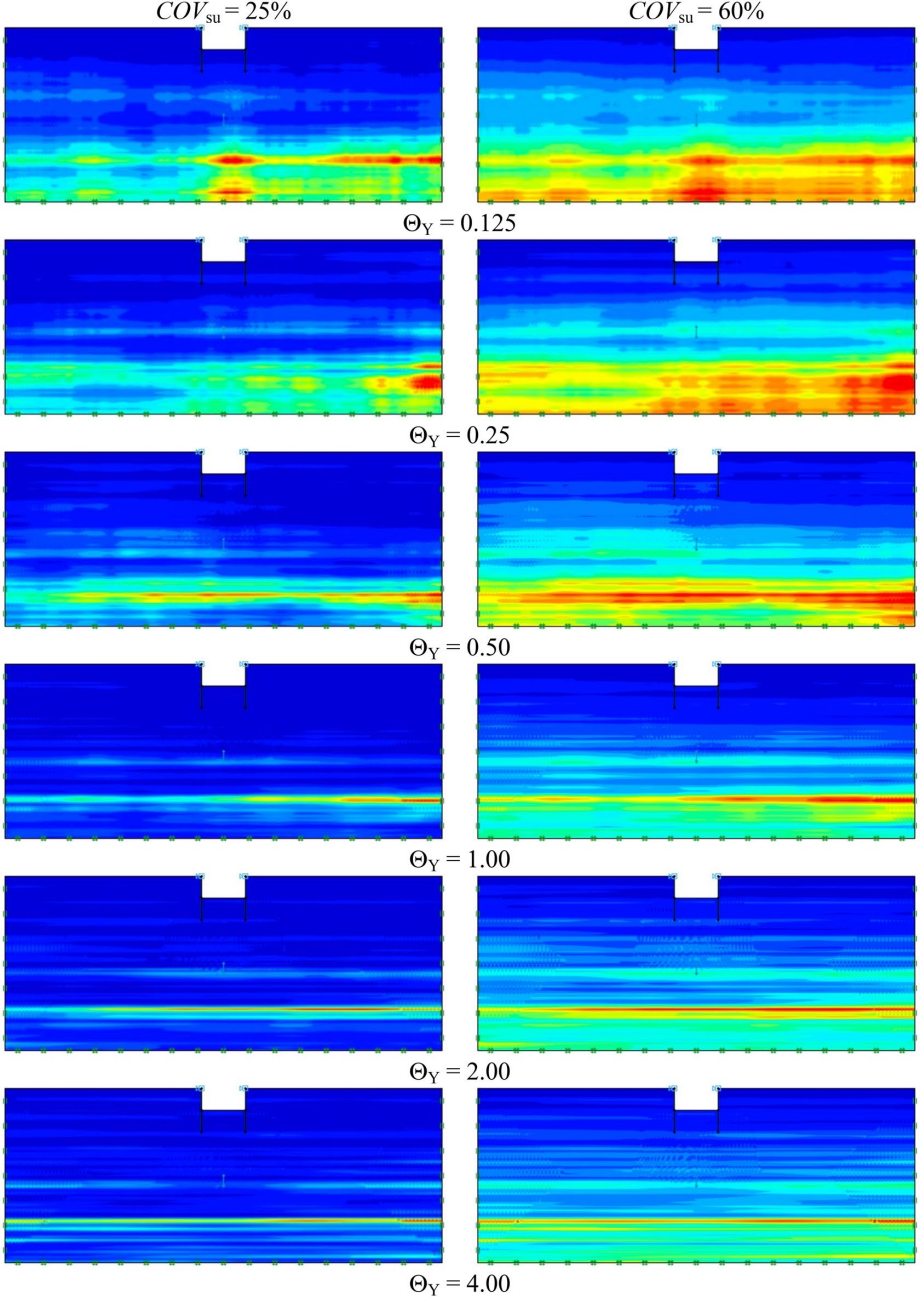
Distribution of undrained shear strength with different normalised correlation length (Θ_Y_).

Examples of the adaptive meshes are shown in Fig. 3 for deterministic analyses and typical realisation of stochastic analyses. The selected values of all parameters in Fig. 3 are *μ*_su0_ = 100; *D* = 1.0 m; *D/H* = 1.0; *B/H* = 4.0; *ρH/μ*_su0_ = 1; *COV* = 60%; Θ_X_ = 50.0; and Θ_Y_ = 1.00. Figure 3 shows that the distribution of the undrained shear strength *s*_u_(*z*) for the deterministic analyses is a linearly increasing profile whereas that of the typical realisation shows a slightly ragged field of *s*_u_(*z*) occurring mainly in the vertical direction due to the moderate value of Θ_Y_ = 1.0. The final adaptive meshes after three steps are also presented in Fig. 3, where the number of meshes increases in the zones that have high plastic shear strains which can reveal the failure patterns of the problem. Figure 4 shows other typical realisations of random fields for undrained shear strength for different values of Θ_Y_ = 0.125 to 4.00 and *COV*_su_ = 25 and 60%. The cases of the greater *COV* values result in higher values of s_u_ and these values decrease with increasing normalised correlation length from Θ_Y_ = 0.125 to 4.00. The distribution of *s*_u_(*z*) with a smaller value of Θ_Y_ is more ragged in the vertical direction of random field profiles.

The current study considers the six design parameters, including *B/H*, *D/H*, *ρH/μ*_su0_, *COV*_su_, Θ_X_ and Θ_Y_. Using the random stability number (*N*_*ran*_) and the associated factors of safety (*FS*), a series of probabilistic design charts are presented for practical design uses and as decision-making considering the uncertainty of soil property. The selected range of all dimensionless parameters is shown in Table 1. Phoon and Kulhawy^51^ indicated that the values of *COV* are about 25% to 60% for characterising inherent variation of soil properties at the site. The value of Θ_X_ is also set to be 50 according to the previous works by Wu et al.^47^ and Wu et al.^48^. The values of *B/H*, *D/H*, and *ρH/μ*_su0_ are set to follow the previous works by Ukritchon et al.^10^ and Keawsawasvong and Ukritchon^11^.

## Results and discussion

**Figure 5:**
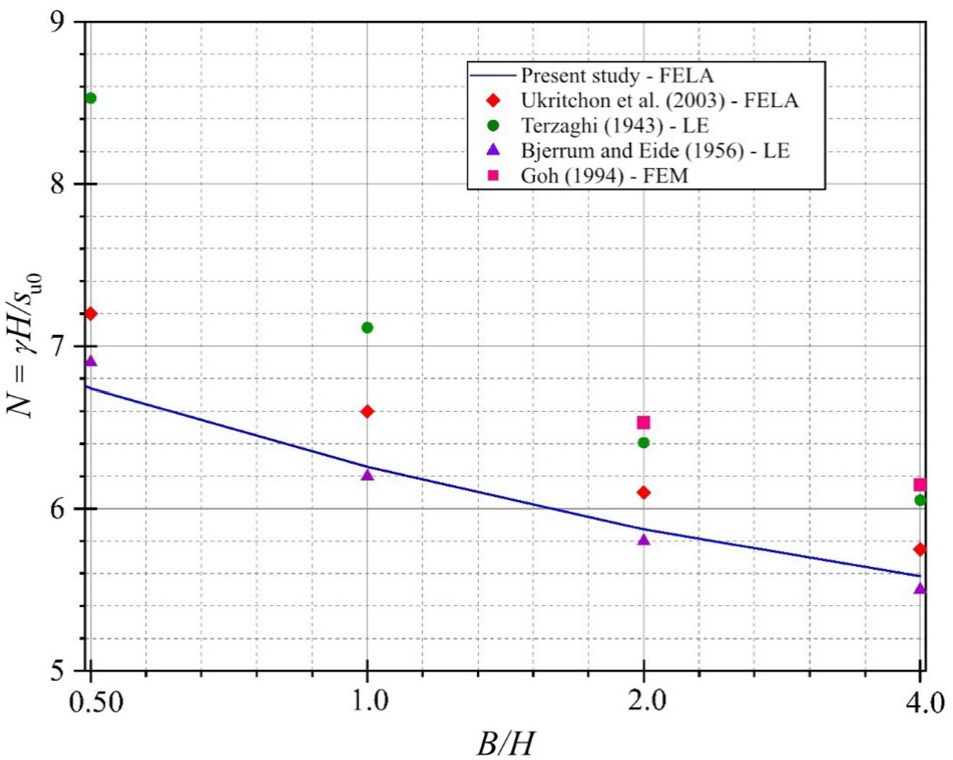
Comparison with previous studies for *D/H* = 0 and *ρH/s*_u0_ = 0.

As the first step of the investigation, the deterministic stability number of fully braced supported excavations in homogeneous clays under plane strain conditions from the present study is compared with existing solutions from the limit equilibrium method (LEM) by Terzaghi^1^ and Bjerrum and Eide^2^, FEM by Goh^3^, and FELA by Ukritchon et al.^10^ Fig. 5 shows that the solutions by Terzaghi^1^ and Goh^3^ provide an overestimation of the stability number. The present solutions using AFELA fairly agree with the previous solutions by Ukritchon et al.^10^ and Bjerrum and Eide^2^. Note that Terzaghi^1^ employed the LEM in this analysis by assuming the failure lines of excavations while Goh^3^ carried out the solutions from FEM models with very coarse mesh distribution. This can lead to the large difference from the present study and Terzaghi^1^ and Goh^3^ since this study used the FELA with the mesh adaptivity approach to obtain more accurate limit state solutions of the excavation problem.

**Figure 6:**
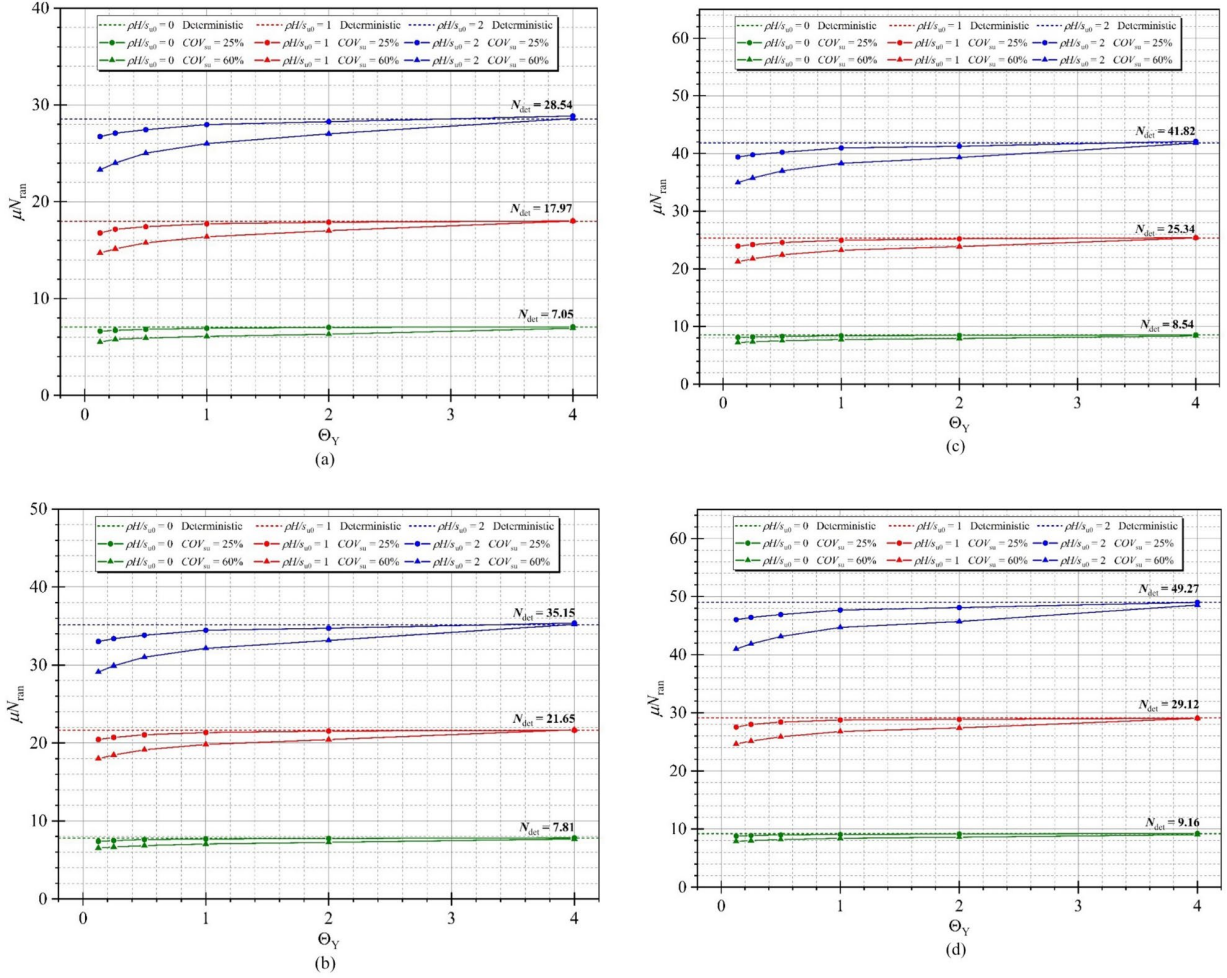
Variation of *μN*_ran_ with different Θ_Y_ for *B/H* = 1.0 and (**a**) *D/H* = 0.25 (**b**) *D/H* = 0.5 (**c**) *D/H* = 0.75 (**d**) *D/H* = 1.0.

All RAFELA results of the mean of random stability numbers *μN*_ran_ are presented in Tables 2 and 3 for COV = 25% and 60%, respectively. Some results are selected to portray the effect of soil strength variability for all considered parameters including width and depth of the excavation ratio, strength gradient factor and dimensionless vertical correlation length. Figure 6 presents the mean stability numbers *μN*_ran_ against the dimensionless vertical correlation length Θ_Y_ for various values of *COV*_su_, *ρH/μ*_su0_, and *D/H* while the value of *B/H* is set to be 1.0. The mean of the random stability number significantly increases and tends to be stable corresponding with the lower and further values of the dimensionless vertical correlation length Θ_Y_ because a lower value of Θ_Y_ causes a rougher spatial variability of *s*_u_. A higher value of Θ_Y_ would lead to a smoother spatial variability of s_u_ dealing with a maintained value of random stability number *N*_ran_. When the value of Θ_Y_ reaches 4.00, the completely smooth random field of soils with linearly increasing strength can be obtained. Hence, the value of the mean stability numbers *μN*_ran_ come closer to the deterministic stability numbers *N*_det_. As expected, different values of the mean stability numbers *μN*_ran_ between *ρH/s*_u_ and COV can be observed, and the higher values of *ρH/s*_u_ and COV are considered. The larger and lower values of the mean stability numbers *μN*_ran_ are also observed.

**Table 2:** The mean of random stability numbers *μN*_ran_ with different Θ_Y_ when *COV*_su_ = 25%.

*COV*_su_	*ρH/μ*_su0_	Θ_Y_	*B/H*																			
			0.25				0.5				1.0				2.0				4.0			
			*D/H*				*D/H*				*D/H*				*D/H*				*D/H*			
			0.25	0.5	0.75	1.0	0.25	0.5	0.75	1.0	0.25	0.5	0.75	1.0	0.25	0.5	0.75	1.0	0.25	0.5	0.75	1.0
**25%**	0	0.125	9.87	12.30	14.67	17.01	7.84	9.15	10.40	11.61	6.63	7.39	8.11	8.79	5.86	6.29	6.72	7.11	5.37	5.60	5.83	6.06
		0.25	9.92	12.41	14.78	17.11	7.96	9.25	10.49	11.73	6.71	7.47	8.19	8.89	5.91	6.35	6.77	7.17	5.39	5.62	5.85	6.08
		0.50	10.05	12.52	14.88	17.19	8.07	9.35	10.63	11.85	6.82	7.61	8.29	8.99	5.97	6.41	6.83	7.23	5.41	5.65	5.89	6.12
		1.00	10.19	12.65	14.99	17.30	8.17	9.46	10.73	11.94	6.93	7.69	8.40	9.06	6.06	6.50	6.93	7.33	5.44	5.69	5.93	6.16
		2.00	10.23	12.69	15.07	17.41	8.26	9.54	10.80	12.01	7.01	7.75	8.47	9.14	6.16	6.60	7.02	7.42	5.51	5.76	6.00	6.23
		4.00	10.25	12.75	15.14	17.47	8.28	9.63	10.89	12.08	7.08	7.83	8.55	9.23	6.31	6.75	7.16	7.54	5.65	5.90	6.13	6.35
	1.0	0.125	22.35	30.07	38.24	46.96	18.56	23.42	28.43	33.73	16.77	20.44	23.93	27.53	16.33	18.98	21.74	24.53	17.75	20.71	23.18	25.56
		0.25	22.62	30.35	38.58	47.30	18.83	23.71	28.78	34.11	17.15	20.72	24.22	28.00	16.46	19.19	21.95	24.76	17.62	20.54	23.03	25.43
		0.50	22.85	30.64	38.88	47.60	19.10	24.02	29.10	34.46	17.42	21.05	24.57	28.40	16.67	19.47	22.26	25.07	17.63	20.50	23.03	25.44
		1.00	23.13	30.95	39.15	47.94	19.40	24.32	29.43	34.85	17.72	21.34	24.94	28.74	16.96	19.81	22.64	25.50	17.79	20.70	23.22	25.71
		2.00	23.15	31.03	39.39	48.27	19.50	24.50	29.77	35.23	17.88	21.53	25.21	28.86	17.24	20.09	22.94	25.83	17.99	20.97	23.53	26.04
		4.00	23.17	31.14	39.54	48.41	19.60	24.64	29.88	35.34	18.01	23.93	25.40	29.06	17.73	20.66	23.56	26.46	18.23	21.23	23.85	26.38
	2.0	0.125	34.66	47.62	61.61	76.69	29.05	37.45	46.41	55.80	26.73	33.02	39.40	46.03	26.22	31.16	36.25	41.42	25.98	31.17	35.92	40.39
		0.25	35.09	48.09	62.16	77.25	29.49	37.92	46.93	56.52	27.09	33.39	39.78	46.41	26.37	31.44	36.56	41.78	25.97	31.11	35.96	40.55
		0.50	35.44	48.55	62.65	77.75	29.91	38.42	47.59	56.96	27.44	33.82	40.23	46.91	26.62	31.84	36.98	42.29	26.10	31.20	36.21	40.93
		1.00	35.88	49.05	63.08	78.31	30.39	39.05	48.10	57.63	27.97	34.46	40.97	47.68	27.22	32.57	37.76	43.05	26.36	31.65	36.69	40.95
		2.00	35.89	49.15	63.46	78.87	30.53	39.25	48.35	58.05	28.27	34.72	41.26	48.10	27.59	32.97	38.28	43.71	26.77	32.64	37.35	40.97
		4.00	35.91	49.32	63.72	79.09	30.67	39.24	48.43	58.00	28.85	35.38	42.13	49.00	28.29	33.79	39.15	44.52	27.31	35.90	38.33	40.99

**Table 3:** The mean of random stability numbers *μN*_ran_ with different Θ_Y_ when *COV*_su_ = 60%.

*COV*_su_	*ρH/μ*_su0_	Θ_Y_	*B/H*																			
			0.25				0.5				1.0				2.0				4.0			
			*D/H*				*D/H*				*D/H*				*D/H*				*D/H*			
			0.25	0.5	0.75	1.0	0.25	0.5	0.75	1.0	0.25	0.5	0.75	1.0	0.25	0.5	0.75	1.0	0.25	0.5	0.75	1.0
**60%**	0	0.125	8.46	11.35	13.65	15.92	6.98	8.28	9.48	10.64	5.50	6.53	7.22	7.88	5.01	5.42	5.82	6.19	4.56	4.78	4.98	5.20
		0.25	8.85	11.59	13.92	16.18	7.15	8.43	9.63	10.78	5.78	6.66	7.35	8.01	5.06	5.49	5.90	6.28	4.57	4.80	5.03	5.25
		0.50	9.29	11.86	14.20	16.42	7.39	8.66	9.88	11.07	5.91	6.84	7.52	8.18	5.18	5.62	6.03	6.42	4.58	4.83	5.07	5.30
		1.00	9.67	12.09	14.42	16.72	7.70	8.97	10.19	11.35	6.09	7.04	7.74	8.40	5.37	5.80	6.21	6.60	4.64	4.90	5.14	5.37
		2.00	9.92	12.32	14.62	16.88	7.91	9.15	10.36	11.52	6.30	7.26	7.93	8.59	5.56	6.01	6.42	6.81	4.78	5.04	5.27	5.50
		4.00	10.34	12.88	15.29	17.67	8.31	9.63	10.54	12.05	6.93	7.68	8.37	9.02	5.91	6.32	6.71	7.07	5.06	5.32	5.54	5.75
	1.0	0.125	21.98	29.7	37.87	46.59	16.61	21.25	25.98	30.89	14.71	17.99	21.26	24.65	13.82	16.25	18.68	21.17	13.98	16.15	18.23	20.26
		0.25	22.25	29.98	38.21	46.93	17.08	21.76	26.65	31.42	15.14	18.46	21.75	25.15	14.01	16.50	18.99	21.53	13.91	16.14	18.30	20.38
		0.50	22.48	30.27	38.51	47.23	17.77	22.50	27.32	32.45	15.76	19.13	22.45	25.88	14.45	17.07	19.63	22.21	14.01	16.26	18.50	20.66
		1.00	22.76	30.58	38.78	47.57	18.56	23.35	28.32	33.46	16.37	19.81	23.24	26.78	15.20	17.84	20.44	23.06	14.29	16.67	18.94	21.31
		2.00	22.78	30.66	39.02	47.9	19.03	23.81	28.74	33.92	17.00	20.44	23.86	27.39	15.83	18.50	21.19	23.96	14.96	17.42	19.81	22.05
		4.00	22.8	30.77	39.17	48.04	19.93	25.02	30.22	35.60	18.01	21.70	25.36	29.06	16.99	19.80	22.53	25.24	15.81	18.45	21.11	23.34
	2.0	0.125	34.29	47.25	61.24	76.32	28.61	37.01	45.97	55.36	23.31	29.12	34.94	41.00	22.17	26.65	31.12	35.69	22.57	27.06	29.35	35.68
		0.25	34.72	47.72	61.79	76.88	29.05	37.48	46.49	56.08	24.01	29.91	35.75	41.88	22.46	27.08	31.63	36.32	22.56	27.00	29.39	35.84
		0.50	35.07	48.18	62.28	77.38	29.47	37.98	47.15	56.52	25.03	31.02	36.98	43.14	23.22	28.00	32.75	37.50	22.69	27.09	29.64	36.22
		1.00	35.51	48.68	62.71	77.94	29.95	38.61	47.66	57.19	26.02	32.15	38.31	44.70	24.43	29.34	34.11	38.98	22.95	27.54	30.12	36.24
		2.00	35.52	48.78	63.09	78.50	30.09	38.81	47.91	57.61	27.02	33.16	39.31	45.71	25.47	30.42	35.37	40.49	23.36	28.53	30.78	36.26
		4.00	35.54	48.95	63.35	78.72	30.23	38.8	47.99	57.56	28.60	35.21	41.81	48.54	27.30	32.61	37.67	42.76	23.90	31.79	31.76	36.28

**Figure 7:**
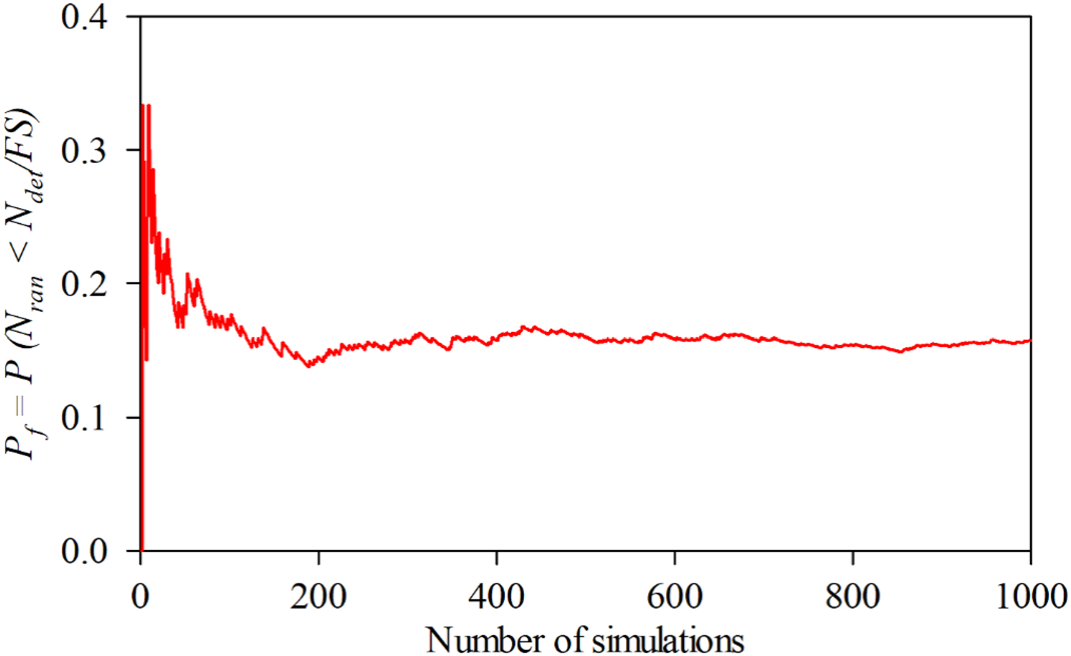
An example of variation of *P*_*f*_ value with number of simulations (A case of *B/H* = 0.25, *D/H* = 1.0, *ρH/μ*_*su0*_ = 1.0, *COV*_*su*_ = 25%, Θ_*Y*_ = 0.5, *FS* = 1.2).

**Figure 8:**
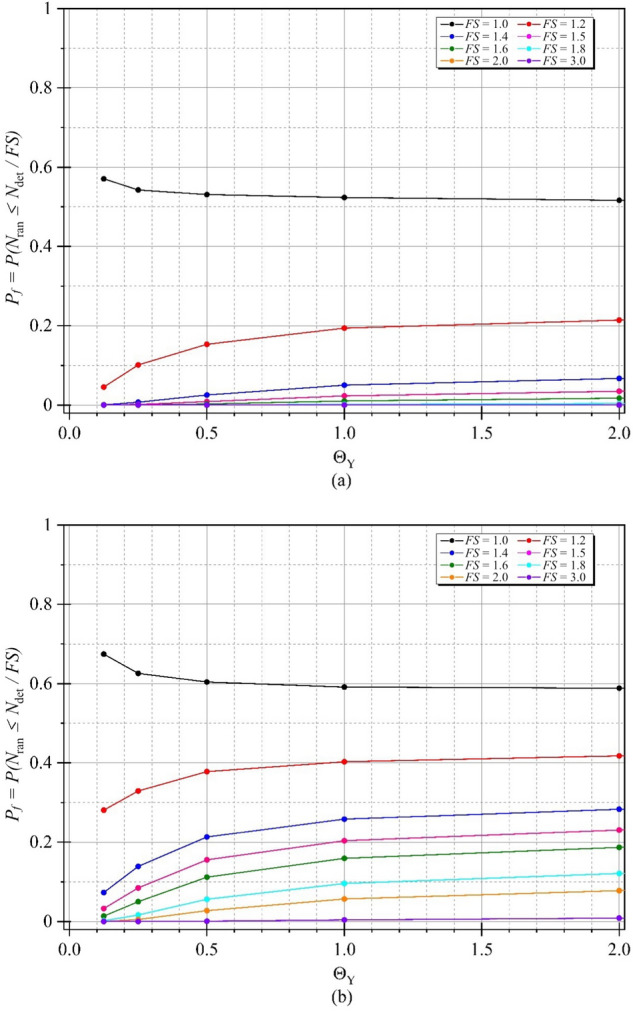
Effect of Θ_Y_ on the probability of failure for *B/H* = 0.25, *D/H* = 1.0, *ρH/μ*_su0_ = 1.0 and (**a**) *COV*_su_ = 25% (**b**) *COV*_su_ = 60%.

**Figure 9:**
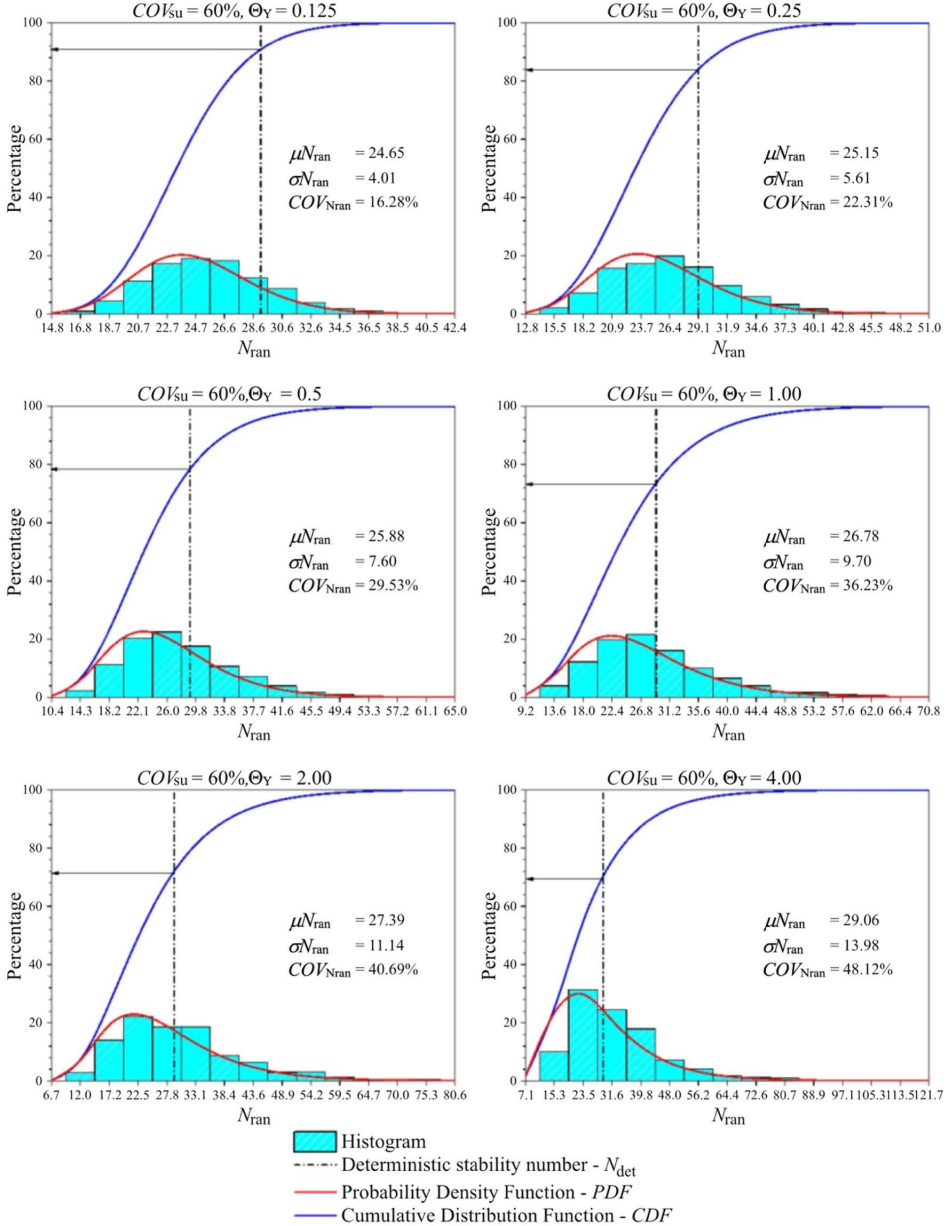
Histogram PDF and CDF for *B/H* = 1.0, *D/H* = 1.0, *ρH/s*_u0_ = 1.0 and *COV*_su_ = 60%.

**Figure 10:**
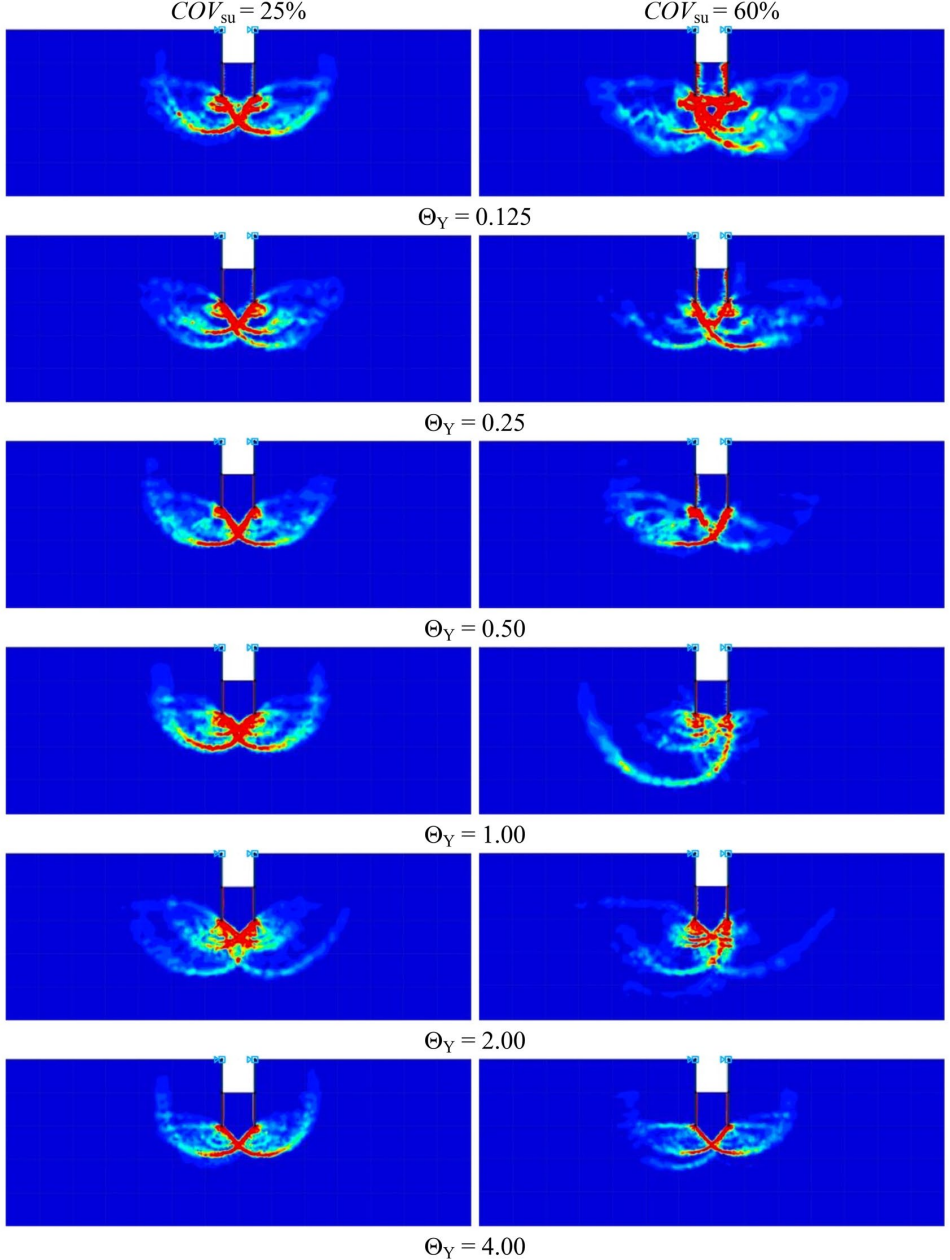
Distributions of shear dissipation for *B/H* = 1.0, *D/H* = 1.0 and *ρH/s*_u0_ = 1.0.

In order to determine accurate probability of design failure (*P*_*f*_), 1,000 simulations were conducted to achieve convergence of *P*_*f*_, ensuring that the *P*_*f*_ has stabilised, which was demonstrated in Fig. 7 as an arbitrary case of braced excavations. Figure 8 shows examples of the variations of the probability of design failure (*P*_*f*_) versus the dimensionless vertical correlation length Θ_Y_, where *B/H* is fixed as 0.25. Note that other parameters in Fig. 8 are *D/H* = 1.0, *ρH/μ*_su0_ = 1.0, and *COV*_su_ = 25% and 60%. The various values of *FS* are considered by varying from *FS* = 1.0 to 3.0. The results indicate the curves are different trends between *FS* = 1.0 and other *FS*. The *P*_*f*_ tends to decrease when *FS* = 1.0, while the increasing values of *P*_*f*_ are generated by *FS* > 1.0 according to increasing values of Θ_Y_ for both *COV* = 25 and 60%. The *FS* = 1.0 is well known as a critical state for simulating stable analysis. In addition, by comparing the results between Figs. 8(a) and 8(b), the effect of *COV*_su_ on the *P*_*f*_ values is found to be significant. A large value of *COV*_su_ can result in a higher value of *P*_*f*_ for the same giving value of *FS*. Moreover, the different *P*_*f*_ among *FS* of *COV* = 60% are higher than that of *COV* = 25%. Based on the results in Fig. 8, the designed *FS* can be derived for no failures. For example, the excavation would be stable during the construction process considering the designed *FS* > 1.6 at any Θ_Y_ and *COV*_su_ = 25%, while the distribution of *s*_u_ is very high fluctuating at the site (*COV* = 60%). Hence, the designed *FS* should be increased, *FS* > 3.0 at any Θ_Y_. From the perspective of modelling material properties and loads in engineering, which are typically positive, the log-normal distribution has the advantage of allowing only positive values. Therefore, the results of all simulations should be considered to fit with the log-normal distribution in this study. The random variable *X* is said to follow a lognormally distribution if *ln*(*X*) follows a normally distribution. The standard deviation, the coefficient of variation and the mean are derived through the transformation of the parameters of the normal distribution as defined in Eqs. (4), (5), and (6). The probability distribution function (*PDF*) and the cumulative distribution function (*CDF*) for the problem of the stability of braced excavations in spatially random clay with different values of Θ_Y_ are presented in Fig. 9. Note that the plots are the manipulation of the 1,000 values of the random stability numbers to develop the histograms in Fig. 9. The probabilistic density function of log-normal distribution is smooth and wide when the values of Θ_Y_ is small. However, the curve of the probabilistic density function becomes narrow and left skewed when Θ_Y_ becomes very larger (e.g., Θ_Y_ = 4.00). The dotted lines in the histograms in Fig. 9 represent the deterministic values of the stability number. The number of random stability numbers *N*_ran_, which are lesser than the deterministic one *N*_det_ (or *FS* < 1), is higher when the value of Θ_Y_ is larger. It should be significantly interesting from the results in Fig. 9 that over half of the number of random stability numbers *N*_ran_ could be observed, which are lower than the value of deterministic analysis even though the spatial variability of *s*_*u*_ becomes to be completely smooth. This implies that ignoring random fields is not suitable for assessing stability excavation. Examples of failure mechanisms of the basal heave stability problem are shown in Fig. 10 for the different values of Θ_Y_ and *COV*_su_. The distributions of shear dissipation are employed to show the pattern of failures, where the values of *B/H* = 1.0, *D/H* = 1.0, and *ρH/μ*_su0_ = 1.0. Figure 10 shows that considering a smaller value of Θ_Y_ and a larger value of *COV*, the failure pattern is not symmetric because of the presence of more generated variation and rough spatial variability of *s*_u_. Figure 10 indicates that the pattern of failures becomes more completely symmetric when the values of Θ_Y_ are larger. The failure patterns of the stochastic case with Θ_Y_ = 4.00 for both *COV*_su_ = 25 and 60% are analogous to that of the deterministic case. The findings in Fig. 10 also demonstrate no differences in the failure patterns between a lower and higher values of *COV* without considering spatial variability of *s*_u_. Thus, the failure mechanisms of the basal heave excavation problem mainly depend on a characterisation of spatial variability of *s*_u_.

**Figure 11:**
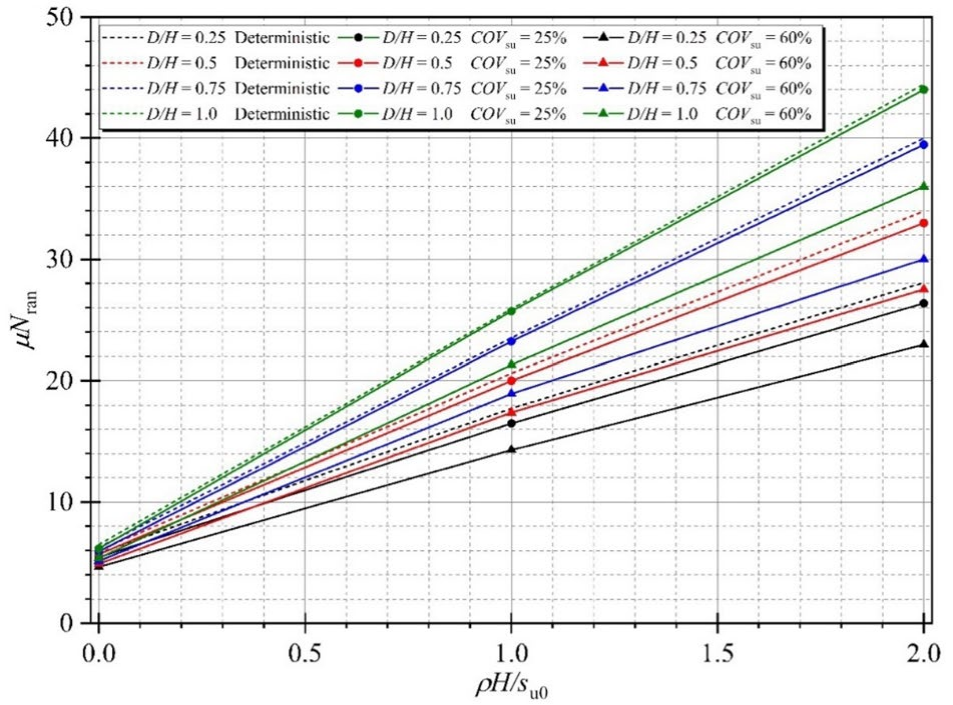
Variation of *μN*_ran_ with different *ρH/μ*_su0_ for Θ_Y_ = 1.0 and *B/H* = 4.0.

**Figure 12:**
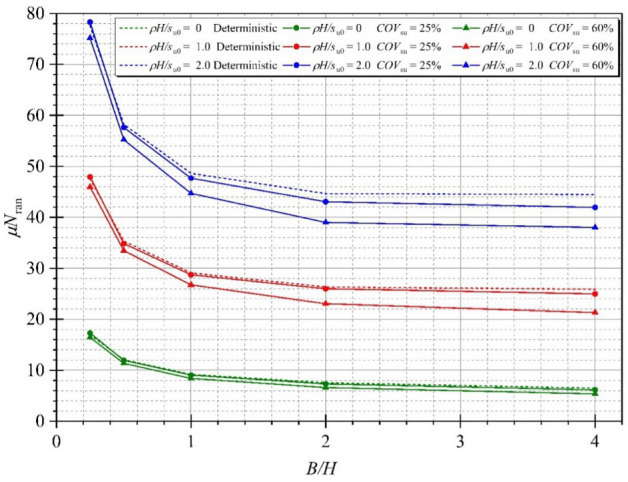
Variation of *μN*_ran_ with different *B/H* for *D/H* = 1.0, *ρH/μ*_su0_ = 1.0 and Θ_Y_ = 1.00.

**Figure 13:**
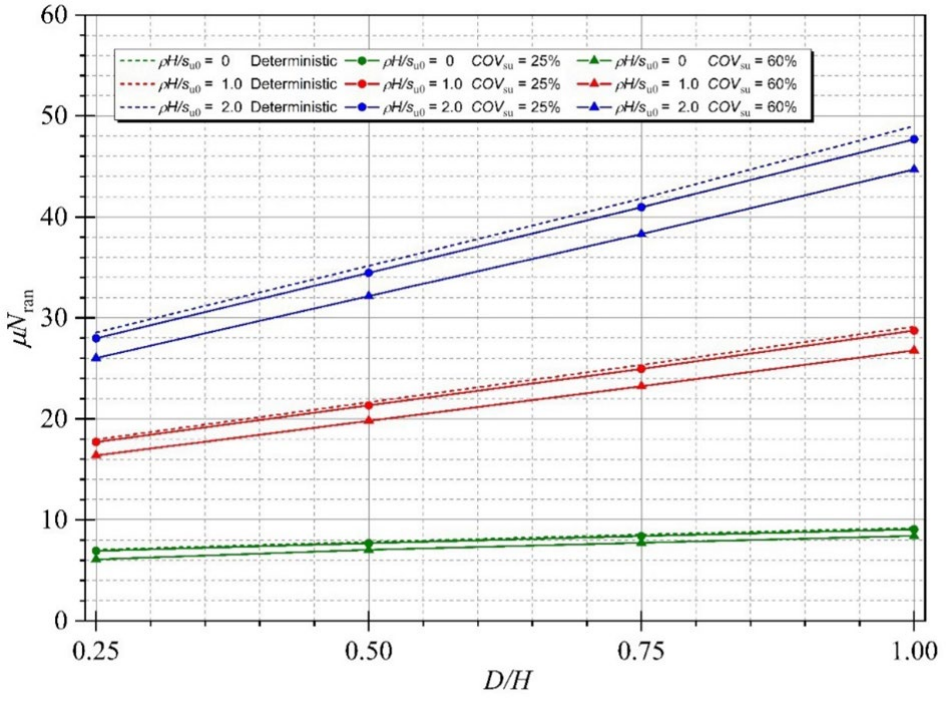
Variation of *μN*_ran_ with different *D/H* for *B/H* = 1.0, *ρH/μ*_su0_ = 1.0 and Θ_Y_ = 1.00.

**Figure 14:**
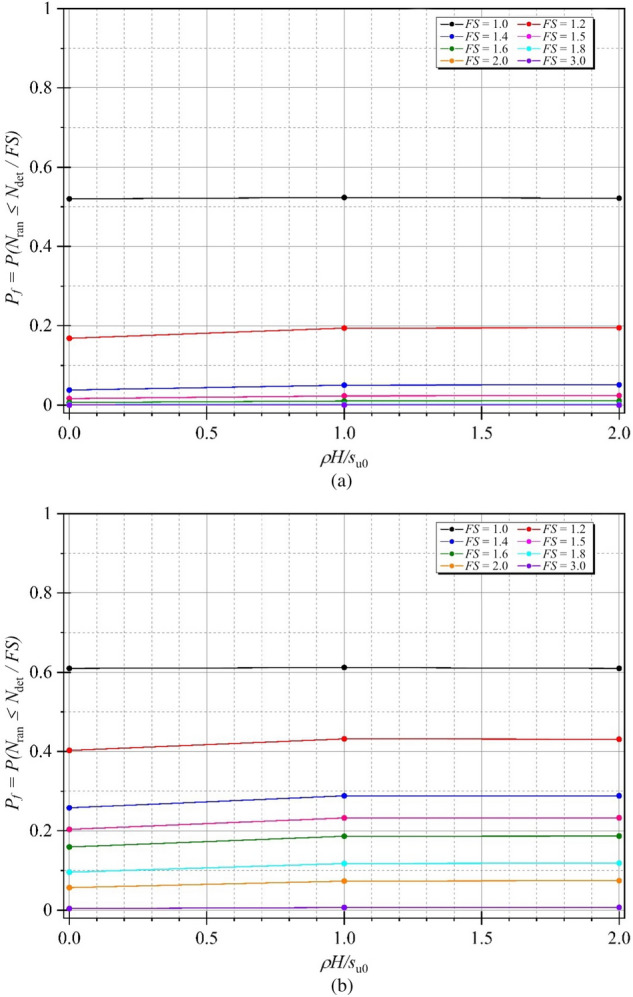
Effect of *B/H* on the probability of failure for *D/H* = 1.0, *ρH/μ*_su0_ = 1.0, Θ_Y_ = 1.00 and (**a**) *COV*_su_ = 25% (**b**) *COV*_su_ = 60%.

The effect of the strength gradient factor *ρH/μ*_su0_ on the mean of random stability numbers is demonstrated next. In Fig. 11, the tendency between the mean stability numbers *μN*_ran_ and *ρH/μ*_su0_ is a linearly increasing line. An increase in *ρH/μ*_su0_ causes an increase in the mean stability number, and the gaps between *COV* = 25 and 60% increase with increasing *ρH/μ*_su0_ because the larger values of the undrained shear strength are simulated, and greater values of random stability number are produced. Figure 12 shows the variation of *μN*_ran_ with different values of the width of excavation ratio *B/H*. Unlike the effect of *ρH/μ*_su0_, the tendency between the mean stability numbers *μN*_ran_ and B/H is the nonlinear decreasing line. The curves of the mean stability numbers *μN*_ran_ quickly go down at the lesser *B/H* (from *B/H* = 0.2 to 2.0) and tend to be flat at the further H/B. The reduced levels of the mean stability numbers *μN*_ran_ are significantly different among *ρH/μ*_su0_ at *B/H* = 0.2 to 2.0, which confirms that an excavation with a high area has less stability than a narrow area. The effects of the depth of excavation ratio *D/H* on the random stability number is illustrated in Fig. 13. The relationship between *μN*_ran_ versus *D/H* is linear, where an increase in the depth of excavation ratio *D/H* yields an increase in the excavation stability. Hence, the level of the excavation stability would be increased corresponding to the larger value of *ρH/μ*_su0_, and remain unchanged at a very small value of *ρH/μ*_su0_ for any *D/H*. Finally, the effect of *B/H* on the probability of design failure (*P*_*f*_) is shown in Fig. 14. The effect of *ρH/μ*_su0_ and *D/H* on *P*_*f*_ is very small and is not presented here for brevity. The results in Fig. 14 have shown that for all chosen values of *FS* from 1.0 to 3.0, an increase in *B/H* causes an increase in *P*_*f*_. When *COV* is larger, the value of *P*_*f*_ is also larger for all giving values of *FS*. Relating to practical applications based on the findings in Fig. 14, the failure probability of braced excavation is not affected by *B/H* if the designed FS is great enough value when the depth of excavation is fixed at the site.

## Conclusion

This study adopted the RAFELA method to investigate the effect of a nonstationary random field of undrained shear strength on the failure probability of fully braced excavation. The quantitative findings from this study can be highlighted as follow:

1. In comparison to the results obtained from the LEM and FEM, the stability number calculated in the present study using the AFELA method was found to be underestimated. Nonetheless, these results align with the deterministic analysis conducted in a previous study utilising the FELA method.
2. In general, the mean stability number of stochastic analysis *μN*_ran_ is smaller than the deterministic stability number. The larger vertical correlation length leads to a further increase in the mean stability number *μN*_ran_ due to the more distributions of smooth spatial variability *s*_u_ were produced by random field theory.
3. Considering probabilistic failure analysis, the more complete failure patterns caused by the smaller vertical correlation length would decrease the probability of design failure. The failure probability was not affected by simulating the higher values of dimensionless vertical correlation length and *B/H*. Besides, the increase in *FS* would drastically decrease the failure probability, it is suggested that the *FS* = 1.6 can guarantee relatively high-level safety braced excavation with *COV*_su_ = 25% for most cases. The *FS* should be raised with the greater *COV*_su_. For example, *FS* = 3.0 with *COV*_su_ = 60% in this study.
4. Based on the statistical analysis of undrained stability in braced excavations, it can be observed that disregarding the spatial variability of undrained shear strength leads to an overestimation of both the mean stability numbers and the probability of designed failures (*P*_*f*_). Therefore, it becomes essential to consider the variability of soil properties for reliable analysis and design of braced excavations, particularly in highly non-homogeneous soil conditions. Moreover, the *P*_*f*_ is highly sensitive to variations in different vertical correlation lengths and the FS.
5. The limitation of this study is that only the 2D plane strain condition is employed in the analysis. Hence, only the stability solutions of 2D braced excavations are considered in the present study, which differs from the real-world case for 3D braced excavations. Future works may include the 3D stability analysis of braced excavations in clay considering the nonstationary random field of undrained shear strength.

## Fundings

The work was supported by Faculty of Engineering Research Fund, Thammasat University and the Thailand Science Research and Innovation Fundamental Fund fiscal year 2023.

## Data Availability

The datasets used and/or analysed during the current study are available from the corresponding author upon reasonable request.
